# The Natural History of Trachoma Infection and Disease in a Gambian Cohort with Frequent Follow-Up

**DOI:** 10.1371/journal.pntd.0000341

**Published:** 2008-12-02

**Authors:** Nicholas C. Grassly, Michael E. Ward, Shirley Ferris, David C. Mabey, Robin L. Bailey

**Affiliations:** 1 Department of Infectious Disease Epidemiology, Imperial College London, London, United Kingdom; 2 Department of Molecular Microbiology, Southampton University Medical School, Southampton, United Kingdom; 3 Department of Infectious and Tropical Diseases, London School of Hygiene and Tropical Medicine, London, United Kingdom; University of California San Francisco, United States of America

## Abstract

**Background:**

The natural history of ocular *Chlamydia trachomatis* infections in endemic communities has not been well characterised and is an important determinant of the effectiveness of different mass treatment strategies to prevent blindness due to trachoma.

**Methodology/Principal Findings:**

A multistate hidden Markov model was fitted to data on infection and active disease from 256 untreated villagers in The Gambia who were examined every 2 weeks over a 6-month period. Parameters defining the natural history of trachoma were estimated, and associations between these parameters, demographic and baseline immune measurements examined. The median incubation period following infection was estimated at 17 days (95% confidence interval: 11–28). Disease persisted for longer than infection (median 21 (15–32) weeks) versus 17 (12–24) weeks), with an estimated median duration of post-infection inflammation of 5 (3–8) weeks. The duration of active disease showed a significant decline with age even after accounting for lower rates of re-infection and disease at older ages (p = 0.004). Measurements of levels of baseline IgA to epitopes in the major outer membrane protein of *Chlamydia trachomatis* were not significantly correlated with protection or more rapid clearance of infection.

**Conclusions:**

The average duration of infection with *Chlamydia trachomatis* especially at younger ages is long. This contributes to the persistence and gradual return of trachoma after community-wide treatment with antibiotics.

## Author Summary

Trachoma is an infectious disease of the eye that causes blindness in many of the poorest parts of the world. In this paper, we use a novel statistical approach to estimate the characteristics of this disease among people living in The Gambia who were examined every 2 weeks over a 6-month period. We found that the typical duration of infection with *Chlamydia trachomatis* and of clinically active disease were significantly longer than previously estimated. We tested different hypotheses about the natural history of trachoma that explain the relationship between infection and disease observed in the field. We also confirmed that disease lasts significantly longer among young children under 5 years old compared with older children and adults, even after accounting for high rates of re-infection in this age group, consistent with the development of immunity with age. The long duration of infection, especially among younger children, contributes to the persistence and gradual return of trachoma after community-wide treatment with azithromycin. This implies the need for high treatment coverage if infection is to be eliminated from a community, even where the return of infection after treatment is seen to be slow.

## Introduction

The scarring and blindness that result from repeated infection of the eye with *Chlamydia trachomatis* represent a significant public health burden in some of the poorest parts of the world [1]. Community-wide treatment with antibiotics can significantly reduce the prevalence of infection and active inflammatory disease [2]–[5] and is central to current efforts led by the World Health Organisation to eliminate blindness due to trachoma by 2020. The effectiveness of different mass treatment strategies depends on several key parameters describing the natural history of trachoma. For example, the duration of infection determines the rate of return of infection after mass treatment and therefore the frequency of treatment rounds needed to achieve a sustained reduction in the prevalence of infection and disease [6]. The development of an effective vaccine against trachoma also requires a better understanding of the natural history of infection, how this changes following prior exposure to infections and the immune mechanisms that effect these changes [7]. The immune response to infection is finely balanced between protective and pathologic components and indeed some early vaccine candidates appeared to increase disease among younger children [8]. Animal models of ocular and genital *Chlamydia* infections have been useful in examining the immune response to infection [9]–[11]. However, inference from animal models is not always appropriate, since elements of the immune response may be host-species specific.

Some information on the progression of infection and inflammatory disease comes from early experimental inoculations of the eyes of volunteers [12]–[16]. However, these studies have been limited by the number of subjects and by the laboratory technology available, and do not provide information about the development of immunity and changes in disease natural history following multiple exposures to infection, such as occurs in endemic communities. Longitudinal cohort studies in trachoma endemic communities are therefore invaluable in providing estimates of the natural history of trachoma and determining immune correlates of protection against infection and disease. Cohort studies of genital chlamydia have identified an important role for interferon-γ in protection against infection [17], and allowed the estimation of the duration of genital infection in asymptomatic women in the absence of antibiotic treatment [18]. However, the long intervals between follow-up visits in this study, and the difficulty in excluding reinfection, lead to considerable uncertainty. In addition genital and ocular strains of *C. trachomatis* show significant differences that can alter their sensitivity to immune effectors, complicating inference between these systems [19].

Here we present an analysis of a cohort study of ocular *C. trachomatis* infection from an endemic community in the Gambia with frequent (two-weekly) follow-up over 6 months [20]. We estimated parameters describing the natural history of trachoma and associations between these parameters and demographic variables and baseline measurements of immunity. The analysis used a multi-state Markov model that allows for re-infection between follow-up visits and permits the sensitivity and specificity of laboratory tests for chlamydia and clinical diagnosis to be estimated.

## Materials and Methods

### Ethical approval

The original study received ethical approval from the joint Gambia Government and Medical Research Council Ethics Committee (SCC 508). All subjects gave oral informed consent that was witnessed and signed by the witness following the standard consent procedures at the time. The current study is an analysis of this original study and did not involve gathering any new information.

### Data

Following a baseline survey 256 individuals from two Gambian villages, Jali and Berending, were examined for clinical and laboratory signs of infection every two weeks over a 6 month period. These individuals were members of 20 households randomly selected from all households in the baseline survey with at least one active trachoma case and at least 3 members without active trachoma. Members of the selected households not present at baseline were included in the survey on their return and thereafter. Clinical scoring of active trachoma was based on the system of Dawson and colleagues [21], and conjunctival swabs tested for the presence of chlamydial lipopolysaccharide antigen by ELISA (IDEIA test, Dako, Copenhagen). Details of the study cohort, clinical and laboratory tests and survival analysis of the data are described elsewhere [20].

At baseline tear fluid IgA and IgG responses to epitopes of the variable segments of the chlamydial major outer membrane protein defined on the basis of their amino acid sequence were measured using ELISA methods to assess antibody binding to epitopic peptide segments conjugated to human serum albumin as previously described [22].

### A simple Markov model of trachoma infection and disease

**Figure 1:**
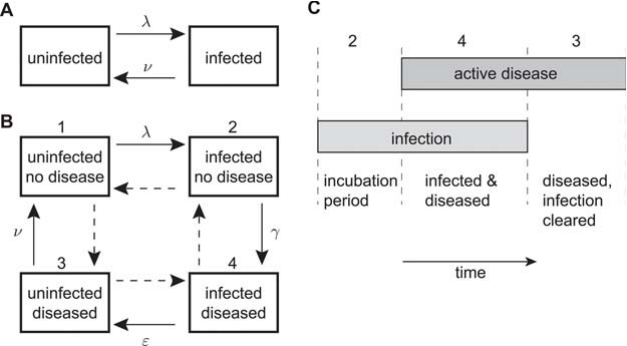
Markov models of the natural history of active trachoma. A) A simple model of infection with a constant hazard of becoming infected *λ* or recovering from infection *ν*. This same two-state model can be used to describe the process of the onset and resolution of inflammatory disease. B) A four state model of infection and disease with states indexed from 1 to 4. In this case transition intensities for the infection and disease processes are mutually dependent. For example, the probability of clearing infection depends on whether inflammatory disease is present. The ‘standard’ model of trachoma natural history corresponds to the solid arrows with transition parameters marked. This is illustrated in C), where time runs from left to right and disease states are indicated at the top of the figure.

To begin with, we considered the cohort data on infection and clinical diagnosis separately. In this case, incident infection (or onset of disease) and the clearance of infection (or disease resolution) in an individual is a simple two-state Markov process with constant hazards *λ* and *ν* respectively over the period of analysis (Figure 1A). A Markov process is one in which the future state of the system depends only on the present state and not the history of the system. Here we assumed active disease includes mild, moderate and severe disease, which encompass both intense trachoma (TI) and follicular trachoma (TF) in the simplified WHO scoring system [23].

In each case, the Markov process can be characterized by the transition intensity matrix
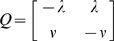
(1)where *q_rs_* describes the instantaneous hazard of moving from state *r* to state *s* for *r*≠*s* and 
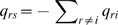
 for *r* = *s* [24]. If two observations of an individual are separated by time *t* , then the probability of observing a transition from *r* to *s* is described by the transition probability matrix *P* with entries *p_rs_*(*t*). This may be calculated from the matrix exponential *P*(*t*) = exp(*tQ*), which, in the case of this simple 2-state Markov process, is given by
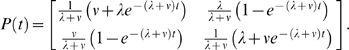
(2)


### A more complex model of the natural history of infection and disease

Where cohort studies include laboratory tests for infection in addition to clinical diagnoses at follow-up visits it is possible to test the fit of different models of disease natural history. In this case we considered a four state Markov model, with each state corresponding to whether an individual is infected and whether they have active disease (Figure 1B). Using this approach, it was possible to test the fit and estimate the parameters of different models of disease natural history. For example, the ‘standard’ natural history summarised by Wright and Taylor [9], and illustrated in Figure 1C, corresponds to a transition intensity matrix
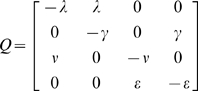
(3)where *q_rs_* describes the instantaneous hazard of moving from state *r* to state *s* (with states given in Figure 1B). Note that we do not allow instantaneous transitions that result in changes in both the infection and disease state, although over a longer period such transitions may be observed, with the probability given by *P*(*t*) = exp(*tQ*) as before.

### Hidden Markov Models

Laboratory and clinical diagnoses are not without error. In the case of enzyme immunoassay for chlamydial antigen the sensitivity *η* and specificity *σ* may be quite low. Observer error can also compromise the sensitivity and specificity of clinical diagnosis, although the magnitude of this effect is likely to be smaller. In other words, the true status of each individual is ‘hidden’ and we only observe the results of the laboratory tests and/or clinical diagnoses. The relationship between the true status of an individual and that observed is given by the emission matrix of the hidden Markov model (HMM). In the two-state model (Figure 1A) this is simply
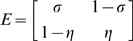
(4)where *e_sw_* is the probability an individual in state *s* has laboratory result (or clinical diagnosis) *w*, and *σ* is the specificity and *η* the sensitivity of the laboratory test (or clinical diagnosis). If *E_l_* is the emission matrix for the infection process and *E_c_* the emission matrix for the disease process, then the emission matrix of the four state model of trachoma natural history is given by the matrix direct product *E_c_*⊗*E_l_*.

### Likelihood of the hidden Markov model

Each individual in a cohort study may be clinically examined and/or tested for infection on a number of occasions. We denote the observation for individual *i* on the *j*th occasion by *y_ij_* and the time of observation by *t_ij_*. The true (hidden) state of individual *i* is given by *x_ij_*. The number of times each individual is observed during a study will depend on the frequency and success of follow-up visits. We assume that these are independent of the underlying infection or disease state of the individual.

The likelihood contribution of individual *i* can be written as a matrix product, following MacDonald and Zucchini [25] and Jackson [26],
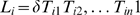
(5)where *δ* is a vector with the *k*th entry equal to the product of the probability that individual *i* initially occupies state *k*, Pr(*s_i_*_1_ = *k*) and the probability of observation *y_i_*_1_ given that underlying state 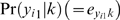, **1** is a column vector consisting of ones, *n* is the number of observations for individual *i* and *T_ij_* is a matrix given by
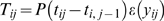
(6)where *ε*(*y*) = diag(*e*_1*y*_, *e*_2*y*_) or diag(*e*_1*y*_, *e*_2*y*_, *e*_3*y*_, *e*_4*y*_) for the 2-state and 4-state models respectively.

### Estimating parameters

The transition hazards and sensitivity and specificity of the laboratory tests (or clinical diagnoses) were estimated from the data by finding the HMM with the maximum likelihood. We also examined whether the transition hazards in the 2-state models depended on covariates including age, sex and baseline immune parameters, using a proportional hazards model, such that the entries of the transition intensity matrix are given by
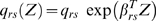
(7)where *β_rs_* is the vector of regression coefficients associated with a vector of covariates *Z* for the transition from *r* to *s* [26],[27]. We assumed that covariates did not change over time and that the sensitivity and specificity of the laboratory tests and clinical diagnoses did not depend on age or change over time. In the 4-state model the sensitivity and specificity of the laboratory tests and clinical diagnoses were fixed at the estimates from the two-state model and age was not considered as a covariate on transition intensities.

The initial probability distribution for the ‘true’ underlying states of individuals at the first observation (the probabilities Pr(*s_i_*_1_ = *k*) that make up *δ* in equation (5)) was estimated by finding the HMM with the maximum likelihood. This distribution was allowed to depend on the covariates of the transition intensities (for example, the baseline prevalence of infection and disease decline with age). The dependence of the baseline infection or disease status on covariates such as age was estimated using multinomial logistic regression since the probabilities must sum to one (see [26] for details).

Asymptotic estimates of the standard error of the estimates of *Q, E*, *δ* and *β* were obtained by inverting the Hessian matrix of the maximized log-likelihood following the methods of Kalbfleisch and Lawless [28]. Approximate confidence intervals about the estimates are given assuming normality on the log-scale for *Q* and *β* and on the logit scale for entries in *E* and *δ*. Plots of the likelihood as a function of pairs of parameter estimates were examined to determine the appropriateness of this assumption. Different (nested) models were compared using the likelihood ratio statistic [24],[29].

To maximise the likelihood and estimate confidence intervals we used the freely available multi-state modelling (msm) package written in the R programming language with the simulated annealing optimization method [26].

### Model fit

The ability of the 2-state HMM to describe the observed data was assessed by simulating observations under the model for infection and the model for active disease with parameters set to their maximum likelihood estimates. For each individual in the cohort 1000 simulated observations were produced based on the reported examination times and with the initial state randomly drawn from the probability distribution estimated from the data. The number of transitions between infected and diseased states in the simulations was then compared to the actual number observed.

## Results

### Duration of *C. trachomatis* infection and active inflammatory disease

The estimated median duration of active trachoma (TI and TF) was approximately 8 months among children 0–4 years old but with broad 95% confidence intervals (Table 1). At older ages the estimated duration of active disease declined to 18.8 weeks for children aged 5 to 14 years old, and to 7.0 weeks for older children and adults. The duration of active disease in older children and adults was significantly shorter than for children aged under 5 years (p = 0.004). The hazard of developing active disease showed a significant decline with age (likelihood ratio = 5.4, p = 0.005; Table 1).

**Table 1:** Duration of trachoma infection and disease, and sensitivity and specificity of laboratory tests and clinical diagnoses (with approximate 95% confidence intervals).

	Sensitivity (%)	Specificity (%)	Age (years)	Median duration (weeks)	Hazard of infection/disease (per year)
*Disease*	97 (95–98)	93 (90–95)			
			0–4	36.1 (17.7–73.5)	1.4 (0.73–2.6)
			5–14	18.8 (11.5–30.7)	0.68 (0.38–1.2)
			15+	7.0 (2.6–19.2)	0.35 (0.15–0.83)
*Infection*	77 (70–82)	94 (92–96)			
			0–4	15.4 (8.3–28.6)	1.3 (0.6–2.6)
			5–14	8.2 (5.3–12.6)	0.58 (0.24–1.4)
			15+	7.6 (2.2–26.9)	0.072 (0.0036–1.4)

The estimated duration and hazard of infection with *C. trachomatis* show a similar pattern with age to that for disease. The duration of infection in children aged 0–4 years was estimated at 15.4 weeks, compared to 8.2 weeks for children aged 5–14 years old and 7.6 weeks for older children and adults, although this decline was non-significant (likelihood ratio = 2.2, p = 0.11). The estimated duration of infection was shorter than the duration of disease at all ages, suggesting persistence of disease for a period after the clearance of infection. The hazard of infection showed a significant decline with age (likelihood ratio = 3.2, p = 0.04).

The hazard of infection or developing active disease was not found to depend on sex. The duration of infection was independent of sex, but there was some evidence of a shorter duration of active disease among males (p = 0.02).

### Sensitivity and specificity of laboratory tests and clinical diagnoses

**Figure 2:**
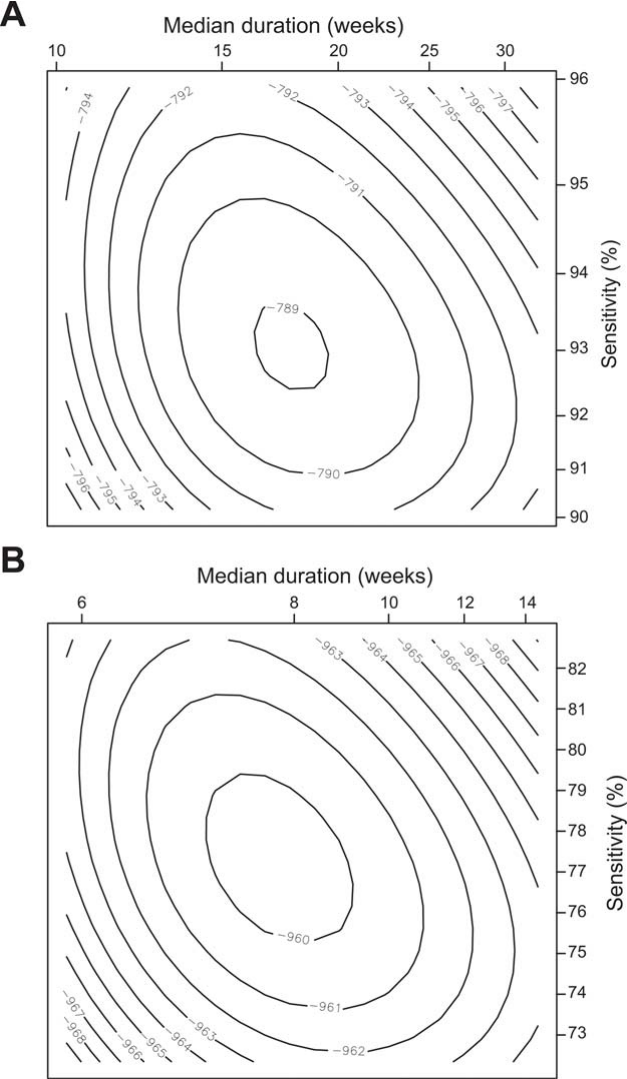
Likelihood of the hidden Markov model for active disease or infection, for different values of the median duration and test sensitivity plotted about the maximum likelihood estimate. The model for active disease is shown in A) and infection in B). In each plot the duration of disease or infection is plotted on a log-scale and sensitivity on the logit scale.

The sensitivity and specificity of the laboratory tests and clinical diagnoses to detect the underlying infection and disease states predicted by the Markov model were estimated at the same time as the duration of infection and disease (Table 1). The maximum likelihood estimates of the sensitivity of the laboratory tests and clinical diagnoses were well-defined and the likelihood surface was consistent with the normality assumption used to generate approximate confidence intervals (Figure 2).

### Natural history of trachoma

The transition intensities of the four-state Markov model corresponding to the ‘standard’ model of trachoma natural history illustrated in Figure 1C were estimated from the data (Table 2). The fit of this four-state model provided an estimate of the median duration of the incubation period of about 2 weeks (95% CI: 11–28 days), and of post-infection inflammatory disease of about 5 weeks (3.6 to 8.0 weeks) in addition to estimates of the duration of infection and active disease.

**Table 2:** Parameter estimates for different models of trachoma natural history using the best estimates of the sensitivity and specificity of tests of infection and disease.

Stage (Markov model state label in Figure 1B)	Median duration and approximate 95% confidence intervals			Time units
	Standard model Log-likelihood = 1743.1	Modified model Log-likelihood = 1736.1	Modified model with active disease sustained by repeat infection Log-likelihood = 1727.9	
Infection (2 & 4)	17.2 (13.7, 23.0)	18.2 (13.9, 26.2)	14.4 (10.3, 20.6)	weeks
Active disease (4 & 3)	21.1 (16.8, 28.4)	23.2 (18.9, 32.5)	26.9 (19.9, 38.4)	weeks
Incubation (2)	17.4 (10.9, 27.7)	15.4 (6.9, 32.8)	20.0 (10.5, 34.8)	days
Post-infection inflammation (3)	5.4 (3.6, 8.0)	5.1 (3.4, 7.7)	3.6 (2.2, 5.5)	weeks

The likelihood of a model that allows for inflammatory disease (TI/TF) due to an immediate cause other than infection with *C. trachomatis* (transition path 1→3→1 in Figure 1B) was significantly better than the standard model (likelihood ratio = 3.6, p = 0.002). The estimated hazard of active disease in uninfected individuals is smaller (0.18 per year) than the hazard of infection with *C. trachomatis* and subsequent disease (0.31 per year). A model that allowed for asymptomatic infection also gave an improved fit compared with the standard model (likelihood ratio = 3.3, p = 0.005). The estimates of the parameters defining the natural history of trachoma were similar in both the standard model and a modified model, in which asymptomatic infections and inflammatory disease without detectable *C. trachomatis* infection occur (Table 2). Further extending the model of natural history to include the possibility of repeated infection leading to sustained active disease (transition path 4→3→4) significantly improved the fit of the model compared with this modified model (likelihood ratio = 8.2, p<0.001; Table 2).

The estimated probability that an individual diagnosed with active disease was infected with *C. trachomatis* (positive predictive value) was 71% based on the fit of the standard model of natural history, 61% in the modified model and 65% in the modified model with active disease sustained by repeat infection. The estimated probability that an individual diagnosed without active trachoma was uninfected (negative predictive value) was 91%, 90% and 91% based on the fit of these models respectively.

### The effect of baseline immunological parameters on trachoma natural history

Elevated levels of tear IgA to previously defined epitopes [22] in the VS1 and VS2 domains of serotypes A and B respectively were found among 55% and 17% of the cohort at baseline (elevated IgA was defined as those individuals with >0.1 adjusted optical density units, consistent with earlier studies [30]). However, elevated IgA was not found to correlate with protection against infection with these subtypes (relative rate (RR) of infection in the 4-state model of 45 (95% CI: 0.21–9670) and 0.92 (0.05–15.5) respectively). Similarly, IgA levels to the same epitopes did not show a significant association with the rate of clearance of infection (transition from 4 to 3).

### Model fit

Simulations under the maximum likelihood HMMs of the infection and disease process resulted in a similar number of transitions to that observed in the cohort (Table 3).

**Table 3:** Number of transitions in disease and infection status observed in actual cohort and simulated data.

Transition	Data	Number*	
From\To		No Disease	Diseased
No disease	Observed	1320	112
	Simulated	1378 (1279, 1465)	105 (87, 125)
Diseased	Observed	112	513
	Simulated	108 (91, 129)	465 (386, 556)
From\To		Uninfected	Infected
Uninfected	Observed	1415	162
	Simulated	1407 (1325, 1489)	183 (159, 189)
Infected	Observed	176	253
	Simulated	167 (143, 189)	248 (194, 305)

*: median, 2.5^th^ and 97.5^th^ percentiles of ranked counts are shown for simulations.

## Discussion

The ‘standard’ model of the natural history of trachoma illustrated in Figure 1 is based largely on observations following inoculation of cynomolgus monkeys with *C. trachomatis* and early experimental inoculations of a small number of volunteers [9], [12]–[16]. This paper presents the first test of this model in an endemic community.

**Figure 3:**
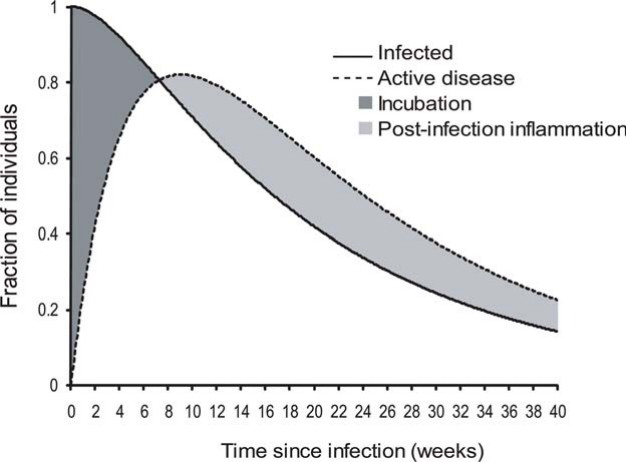
Development of active disease (TF/TI) after ocular infection with *C. trachomatis* estimated from cohort data under the standard model of trachoma natural history. The fraction of individuals who remain infected (solid line) or who have active disease (dashed line) is plotted against the time since infection. The shaded areas highlight individuals with incubating infections or post-infection inflammation (active disease). The parameter estimates are from Table 2.

After ocular infection with *C. trachomatis*, active inflammatory disease develops rapidly and persists for some time after infection has been cleared (Figure 3). The estimate of the inflammatory period after disease clearance (Table 2) is consistent with the observation in a clinical trial of a median duration of active trachoma of about 5 weeks after treatment of infection with azithromycin [31]. The persistence of disease predicts a significant prevalence of active disease without detectable *Chlamydia* organisms, in agreement with observations from cross-sectional studies [32]–[36]. Thus active disease is not strongly predictive of infection (positive predictive value 61%–71%) and so targeting diseased individuals for treatment will result in a significant number of unnecessary treatments (although less than community-wide treatment).

The short estimated incubation period (Table 2) is in reasonable agreement with an average observed incubation period of 6 days (range 2–13 days) for volunteers inoculated with *C. trachomatis* [14]. This suggests that few individuals will have infection detectable by enzyme immunoassay without active disease. This remains the case even after allowing for asymptomatic infections in the modified model, which finds the absence of active disease is strongly predictive of the absence of infection (negative predictive value 90%). This may be helpful in excluding communities with a low prevalence of active disease from treatment. Recent studies support this conclusion, describing a high prevalence of active disease among individuals with active (high-load) infections based on quantitative PCR for chlamydial RNA or DNA [32],[37],[38]. This clarifies the interpretation of earlier studies that used qualitative PCR to detect chlamydial DNA and found conjunctival swabs from disease-free individuals often tested positive [33]–[36]. Whilst some of these positive tests may be early infections prodromal to inflammatory disease, frequent detection of low levels of chlamydial DNA may also reflect inoculation of the eye with non-viable organisms or cross-contamination during examination [9],[37].The presence of infection without a diagnosis of active disease may also occasionally reflect problems with clinical grading.

Overall, infection with *Chlamydia trachomatis* is estimated to last a median of about 17 weeks and active disease (TI/TF) somewhat longer at about 21 weeks in the standard model (Table 2). These estimates are longer than previously estimated from the same cohort study, since they allow for the possibility of false negative and false positive tests that can break up long runs of positive or negative tests or diagnoses. Traditional survival analysis does not allow for this process, leading to an under-estimate of the duration of infection and disease, and an over-estimate of the hazard of infection (this can be illustrated by fitting the Markov model with 100% test sensitivity and specificity; Table S1). The longer duration of infection compared to previous estimates from the same dataset [20] means that infection will emerge more slowly after mass treatment than expected from baseline prevalence if the duration of infection were shorter. However, it also means infection can persist at low levels, highlighting the importance of achieving high coverage during community-wide antibiotic treatment. Slow rates of return of infection and in some cases local elimination have been observed in low to moderate prevalence communities following treatment [2],[4],[39].

The estimated duration of ocular *Chlamydia trachomatis* infection is shorter than that found for female genital infections, where infection typically last for a median of about one year [18], [40]–[42] (although re-infection between infrequent follow-up visits could not be excluded in these studies). This may be the result of the better ability of *C. trachomatis* to persist in the genital tract compared with the conjunctiva.

Previous analysis of the cohort data examined in this study found a significant decline in the duration of infection and disease with age suggesting the importance of acquired immunity to trachoma [20]. It has been suggested that the apparent decline in duration with age may be the result of less frequent re-infection between follow-up visits rather than evidence for an acquired immune response [43]. However, in the present analysis, this decline remains after accounting for re-infection and misclassification, and is statistically significant in the case of active disease (Table 1). This confirms the importance of acquired immunity at least in the rate of resolution of active inflammatory disease. The estimated hazard of infection and disease also decline with age, consistent with changing exposure, hygiene practices and perhaps acquired immunity to re-infection.

Elevated levels of IgA at baseline to epitopes in the VS1 and VS2 domains of the major outer membrane protein were not found to be associated with a protective effect against infection with *Chlamydia* or with the speed with which infection was cleared. This is in agreement with an earlier follow-up study in the same population [30]. This may be the result of low statistical power or the potential importance of other epitopes and immune mechanisms in protection against infection and disease resolution [44].

Extending the standard model of trachoma natural history to include asymptomatic infection and inflammatory disease in the absence of detectable *C. trachomatis* infection provides a significantly better fit to the observed data. Including the possibility that active disease is sustained by repeated infection before the resolution of disease further improves the fit of the model. The estimates of the key parameters describing the natural history of trachoma are reasonably robust to these extensions (Table 2). Allowing for repeated infection and sustained active disease increases the median duration of active disease, and decreases the median duration of infection and post-infection inflammation. The presence of active disease without *C. trachomatis* infection lowers the positive predictive value of disease for infection. Inflammatory disease in the absence of *C. trachomatis* may be the result of infection with other bacterial pathogens or mechanical irritation due to inturned lashes or a dry ocular surface among individuals with earlier trachoma disease [45].

The estimated sensitivity (77%) and specificity (94%) of the chlamydial lipopolysaccharide immunoassay to detect the infection state predicted by the Markov model are similar to, but somewhat lower than, estimates of 84% and 99% respectively, based on comparison of a fixed (dipstick) version of the assay with PCR results in Tanzania [46]. This may reflect differences in the handling of samples and the potential for cross-contamination, or perhaps the use of a PCR-based test as the gold standard for the dipstick assay [46], where false positive PCR results could raise the estimated specificity of the assay.

The estimated sensitivity (97%) and specificity (93%) of the clinical diagnosis to detect the disease state predicted by the Markov model are greater than for the immunoassay for infection. This agrees with the low estimated observer error in this cohort study of about 2%, based on a repeat examination of 123 individuals on the same day by the same examiner [20]. The observer error does not account for transient inflammatory disease that might result from causes other than *C. trachomatis* infection, and indeed the estimated specificity of clinical diagnosis is lower (93%) than that predicted by an observer error of just 2%.

The constant transition intensities of the Markov model imply that the time spent in each state follows the exponential distribution, and thus that the probability distribution describing the time from entering one state to exiting a subsequent state is a sum of exponential variables (i.e. a gamma distribution). This distribution is quite flexible and is illustrated for the infectious and diseased states in the 4-state model in Figure 3. The resulting estimate of the duration of active disease is in agreement with the estimate based on examining disease in isolation (Table 1). The average duration of infection estimated in the 4-state model is slightly longer than in the 2-state model, which may indicate the role of active disease in providing information about the presence of infection, in addition to the laboratory test, in the 4-state model. The Markov assumption seems reasonable, since simulations of the infection and disease process result in similar numbers of state transitions to that observed in the cohort data (Table 3).

Further cohort studies of trachoma are required to allow the identification of the components of protective immunity against ocular *Chlamydia trachomatis.* The methods described in this paper, based on hidden Markov models, allow for continued exposure to infection and provide estimates of the sensitivity and specificity of clinical and diagnostic tests. These methods are well suited to the analyses of future cohort studies of trachoma.

## Supporting Information

Table S1 Duration of trachoma infection and disease, assuming 100% sensitivity and specificity of laboratory tests and clinical diagnoses (with 95% confidence intervals)
(0.03 MB DOC)
