# Supplementary material for: The Natural History of Trachoma Infection and Disease in a Gambian Cohort with Frequent Follow-Up
Source: PLoS Negl Trop Dis. 2008 Dec 2;2(12):e341. doi: 10.1371/journal.pntd.0000341 (PMC2584235; doi:10.1371/journal.pntd.0000341)
Supplement: Table S1 — Duration of trachoma infection and disease, assuming 100% sensitivity and specificity of laboratory tests and clinical diagnoses (with 95% confidence intervals) (0.03 MB DOC) [file pntd.0000341.s001.doc]

**Supplementary Table 1** Duration of trachoma infection and disease, assuming 100% sensitivity and specificity of laboratory tests and clinical diagnoses (with 95% confidence intervals)

|  | **Age (years)** | **Median duration (weeks)** | **Hazard of infection/ disease (per year)** |
| --- | --- | --- | --- |
| *Infection* |  |  | |
|  | 0-4 | 3.2 (2.4 - 4.1) | 6.2 (4.7 - 8.1) |
|  | 5-14 | 2.1 (1.7 - 2.7) | 4.3 (3.4 - 5.5) |
|  | 15+ | 1.3 (0.9 - 1.8) | 2.1 (1.4 - 3.1) |
| *Disease* |  |  | |
|  | 0-4 | 9.8 (7.1 - 13.7) | 4 (2.9 - 5.5) |
|  | 5-14 | 7 (5.4 - 9) | 2.3 (1.7 - 3) |
|  | 15+ | 1.8 (1.2 - 2.7) | 1.5 (1 - 2.2) |
